# Supplementary material for: Identification of photoperiod-induced specific miRNAs in the adrenal glands of Sunite sheep (Ovis aries)
Source: Front Vet Sci. 2022 Jul 22;9:888207. doi: 10.3389/fvets.2022.888207 (PMC9354845; doi:10.3389/fvets.2022.888207)
Supplement: Supplementary file 1 [file Data_Sheet_1.ZIP › Supplementary file/miRDeep2 file.zip!/miRDeep2 file/Know_Structure/SP42A3/oar-mir-410.pdf]

miRBase precursor : oar-mir-410
Total read count : 497
oar-miR-410-5p read count : 0
oar-miR-410-3p read count : 495
remaining reads : 2

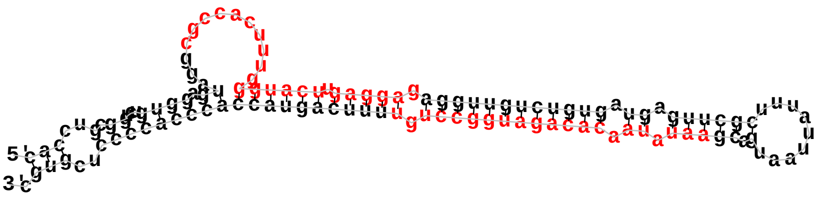

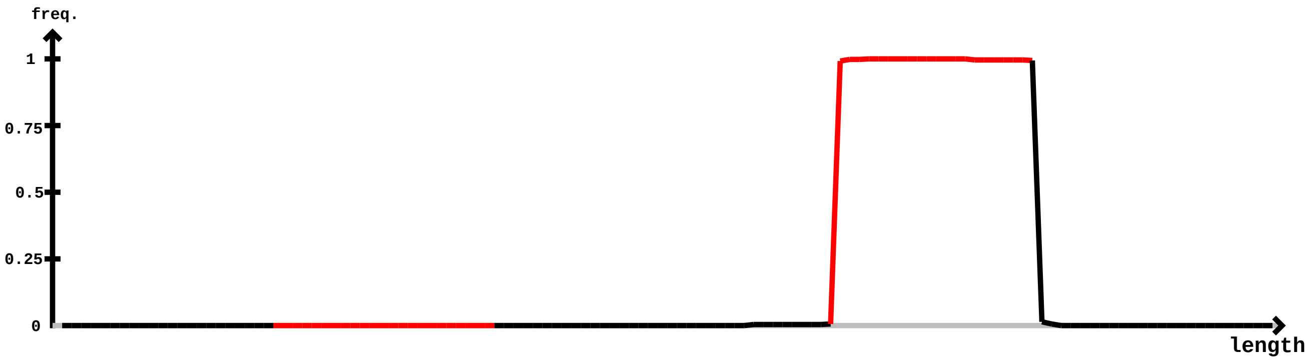


```
                                  oar-miR-410-5p
                                                                        oar-miR-410-3p
5'- caccugcggucuguggaguaggcgccacuuuggguacuugaggagagguugucugugaugaguucgcuuuauuaaugacgaauauaacacagauggccuguuuucaguaccacccaccccucgugc  -3'  exp
    (((..(.(((..((((........))))....((((((.(((((.((((((((((((.((.((((((.........).))))).)).))))))))))))).)))))))))))....))).)..))).   reads  mm
    .......................................................................uuaaugacgaauauaacacagau................................   2      0
    .......................................................................................gaauauaacacagauggccugu.........................   1      0
    ................................................................................aauauaacacagauggccug..........................   1      0
    ................................................................................aauauaacacagauggccugu.........................   484    0
    ................................................................................aauauaacacagauggccuguu........................   4      0
    ................................................................................aauauaacacagauggccuguuu.......................   1      0
    .................................................................................auauaacacagauggccugu.........................   2      0
    .................................................................................auauaacacagauggccuguuu.......................   1      0
    ...................................................................................auaacacagauggccuguuu.......................   1      0
```
